# Trajectories of Depressive Symptoms Among Older Adults and in Adults With Hip Fracture: Analysis From the English Longitudinal Study of Ageing

**DOI:** 10.1093/gerona/glac182

**Published:** 2022-09-08

**Authors:** Rhian Milton-Cole, Salma Ayis, Matthew D L O’Connell, Toby Smith, Katie Jane Sheehan

**Affiliations:** Department of Population Health Sciences, School of Life Course and Population Sciences, King’s College London, London, UK; Department of Population Health Sciences, School of Life Course and Population Sciences, King’s College London, London, UK; Department of Population Health Sciences, School of Life Course and Population Sciences, King’s College London, London, UK; Faculty of Medicine and Health Sciences, University of East Anglia, Norwich, UK; Department of Population Health Sciences, School of Life Course and Population Sciences, King’s College London, London, UK

**Keywords:** Depression, Depressive symptoms, Hip fracture, Trajectories

## Abstract

**Background:**

This study aimed to determine trajectories of depressive symptoms among older adults in England, overall and for those with hip fracture. The study aimed to explore the differential characteristics of each trajectory identified.

**Methods:**

Analysis of adults aged 60 years or more (*n* = 7 050), including a hip fracture subgroup (*n* = 384), from the English Longitudinal Study of Ageing. Latent class growth mixture modeling was completed. Depressive symptom prevalence was estimated at baseline. Chi-square tests were completed to compare baseline characteristics across trajectories.

**Results:**

Three trajectories of depressive symptoms (no, mild, and moderate-severe) were identified overall and for those with hip fracture. The moderate-severe trajectory comprised 13.7% and 7% of participants for overall and hip fracture populations, respectively. The proportion of participants with depressive symptoms in the moderate-severe trajectory was 65.4% and 85.2% for overall and hip fracture populations, respectively. Depressive symptoms were stable over time, with a weak trend toward increasing severity for the moderate-severe symptom trajectory. Participants in the moderate-severe symptom trajectory were older, more likely to be female, live alone, and had worse health measures than other trajectories (*p* < .001).

**Conclusions:**

Older adults, and those with hip fracture, follow one of the 3 trajectories of depressive symptoms that are broadly stable over time. Depressive symptoms’ prevalence was higher for those with hip fracture and, when present, the symptoms were more severe than the overall population. Results suggest a role of factors including age, gender, and marital status in depressive symptom trajectories.

“Late-life depression” is the term used for depression symptomology experienced by older adults (1). Some studies report a positive, linear relationship between age and presence of depressive symptoms where symptoms continually rise after the age of 60 (2). Yet other studies suggest younger older adults have worse depressive symptoms compared with the oldest older adults (3). These differing findings may be attributed to the influence of other demographic and clinical factors such as sex, race, multimorbidity, and life experiences such as spousal bereavement (4). Little is known about the role of these factors on trajectories of depressive symptoms in older adults (4).

Unanticipated health care events such as hip fracture may negatively influence trajectories of depressive symptoms in older adults (4). Cristancho et al. (5) investigated trajectories of depressive symptoms in the year after hip fracture noting 3 distinct trajectories. Investigating whether trajectories differ from the overall older adult population without hip fracture or beyond 1-year postfracture would offer additional understanding of the role of hip fracture in depressive symptoms over time. Furthermore, trajectories of depressive symptoms for older adults with hip fracture may vary between U.S. and UK populations due to demographic and societal differences leading to disparities in health outcomes (6) as well as differences in access to mental health services after fracture (7).

This study aims to determine the trajectories of depressive symptoms among older adults in England over a 17-year period and whether these trajectories vary for those with hip fracture. This study will also explore the differential characteristics of each trajectory identified.

## Method

### ELSA Sample

Participant data for this study were obtained from the English Longitudinal Study of Ageing (ELSA) data set, a nationally representative longitudinal study of community-dwelling adults aged 50 years and older (8). Data on family, work, economic status, physical and mental health, and social, psychological, and biological factors are collected at each wave (9). Participants are followed-up every 2 years with the first wave of data collected in 2002 and 2003. The latest wave, Wave 9, was collected between 2018 and 2019. Ethical approval for ELSA was given by the London Multi-Centre Research Ethics Service (MREC/01/2/91) and written informed consent obtained from all participants. Anonymized unlinked data for this study were provided by the UK Data Service.

### Participants

The analysis cohort for this study (ELSA Waves 1–9) were over the age of 60 years in the year they entered the ELSA sample, with recorded 8-item Centre for Epidemiological Studies-Depression (CES-D) data in at least 2 waves (10), and CES-D scores reported on their exit wave. From all waves, including the refreshment samples, 7 050 people were included for data analysis.

### Measurements

Depressive symptoms were measured using the 8-item CES-D scale (10). The scale has been validated in the older adult population and is a reliable (Cronbach’s α coefficient = .72) and valid tool, with satisfactory model fit (adjusted chi-square test = 0.054, root mean square error of approximation = 0.01, and weighted root mean square residual = 0.63) (11). Each question has a binary response of “yes” or “no,” with a total score of 4 considered the threshold for the presence of depressive symptoms (12).

Alcohol consumption, social networks, Control Autonomy Self-realization Pleasure-19 (CASP-19) (13), and the Life Satisfaction Scale were collected by questionnaire. Age, sex, marital status, health and illness impact, self-rated general health, mobility, comorbidities, activities of daily living (ADL), instrumental activities of daily living (IADL), falls, hip fracture status, overall pain, smoking history, and physical activity were collected during face-to-face interviews. Comorbidities were classified according to 7 ICD comorbidity classifications (14); diseases of the circulatory system (coronary heart disease, angina diagnosis, heart attack, congestive heart failure, other heart disease, or high blood pressure), diseases of the respiratory system (chronic lung disease or asthma), diseases of the nervous system (stroke, Parkinson’s disease, or Alzheimer’s disease), diseases of the musculoskeletal system or connective tissue (osteoarthritis. rheumatoid arthritis, other kind of arthritis, osteoporosis), metabolic diseases (diabetes), mental, behavioral, or neurodevelopmental disorders (dementia) and neoplasms (cancer).

### Statistical Analysis

Analyses were completed for the entire population and for the subgroup of older adults reporting a hip fracture at any wave. This approach was taken due to the potential of an unexpected health care event such as a hip fracture leading to different trajectories of depressive symptoms over time (15). Baseline characteristics stratified by the presence of depressive symptoms were reported as frequencies and percentages.

Group-based trajectory modeling (GBTM), a type of latent class growth mixture modeling application, group individuals by estimating latent trajectories in a population (16). GBTM was conducted to assess unobserved groups of participants following distinct trajectories of depressing symptoms (16,17) using the Stata “traj” and “trajplot” plugins with a censored normal distribution (cnorm) specification (18). “Year of interview” was the time variable and total CES-D scores the variable of interest to identify groups with distinctive patterns of progression.

We tested a series of models using either 2, 3, 4, or 5 groups and combined different polynomial shapes; 0 = intercept, 1 = linear, 2 = quadratic, 3 = cubic. To determine the model of best fit and the optimal number of trajectories, we adopted several model fit indices; the Bayesian information criteria (BIC) (19), entropy (20), the posterior probabilities (21), ensuring a meaningful composition of each trajectory and trajectory membership included at least 5% of data (21). Across all combinations analyzed, we identified the group with the lowest BIC value, the closest entropy value to one, and particularly values over 0.8, which indicate the groups are highly discriminating (19). We also assessed the posterior probabilities of classification in each group. Models in which the average probability of a person being assigned to their assigned group were above 0.8 were more desirable (21). The BIC, entropy, and posterior probabilities for selected models are available in Supplementary Tables S1–S6.

For the overall sample, data were used from the individual’s entry wave. For the hip fracture subgroup, data were used from the wave which included the first reported hip fracture (fracture wave). The proportion of patients with depressive symptoms (numerator as the number of participants whose CES-D ≥ 4 and denominator as the number of participants in each group) at baseline (entry wave) was calculated overall and for each trajectory. Differences between trajectory characteristics were assessed with the Chi-square test (22). Chi-square testing was chosen a priori to assess whether there were differences in characteristics between the trajectories identified. We decided against further post hoc comparisons to identify where the differences lay between each trajectory due to the risk of spurious associations with multiple testing (23). To further limit this risk, we calculated the Bonferroni adjustment which yielded an α level of significance of .0015 (Supplementary Material) (23).

The trajectory analysis method employed automatically imputes missing data for individuals with depressive symptoms measured at least twice. Supplementary Tables S7–S8 display the summaries and patterns of missingness in the data. To assess the sensitivity of findings to missing data, we replicated the analysis excluding cases with missing data and applied the final trajectory model obtained to the first 5 follow-up time points, where the number of missing data was comparatively much lower than the 9 time points used in the main analysis (Supplementary Figures 1–4). Stata 16.1 was used for all analyses (24).

## Results

### Patient Characteristics

Overall, 7 050 patients over the age of 60 were included in the ELSA data set in England between 2002 and 2019 (Supplementary Table S9). Of these, 1 059 (15.0%) patients experienced depressive symptoms.

Characteristics of the study cohort are detailed in Supplementary Table S9. Older adults with depressive symptoms were older, female, more likely to be single, separated, divorced, or widowed, or reported worse health and social outcomes than those without depressive symptoms (Supplementary Table S9).

### Trajectory Analysis

**Figure 1.**
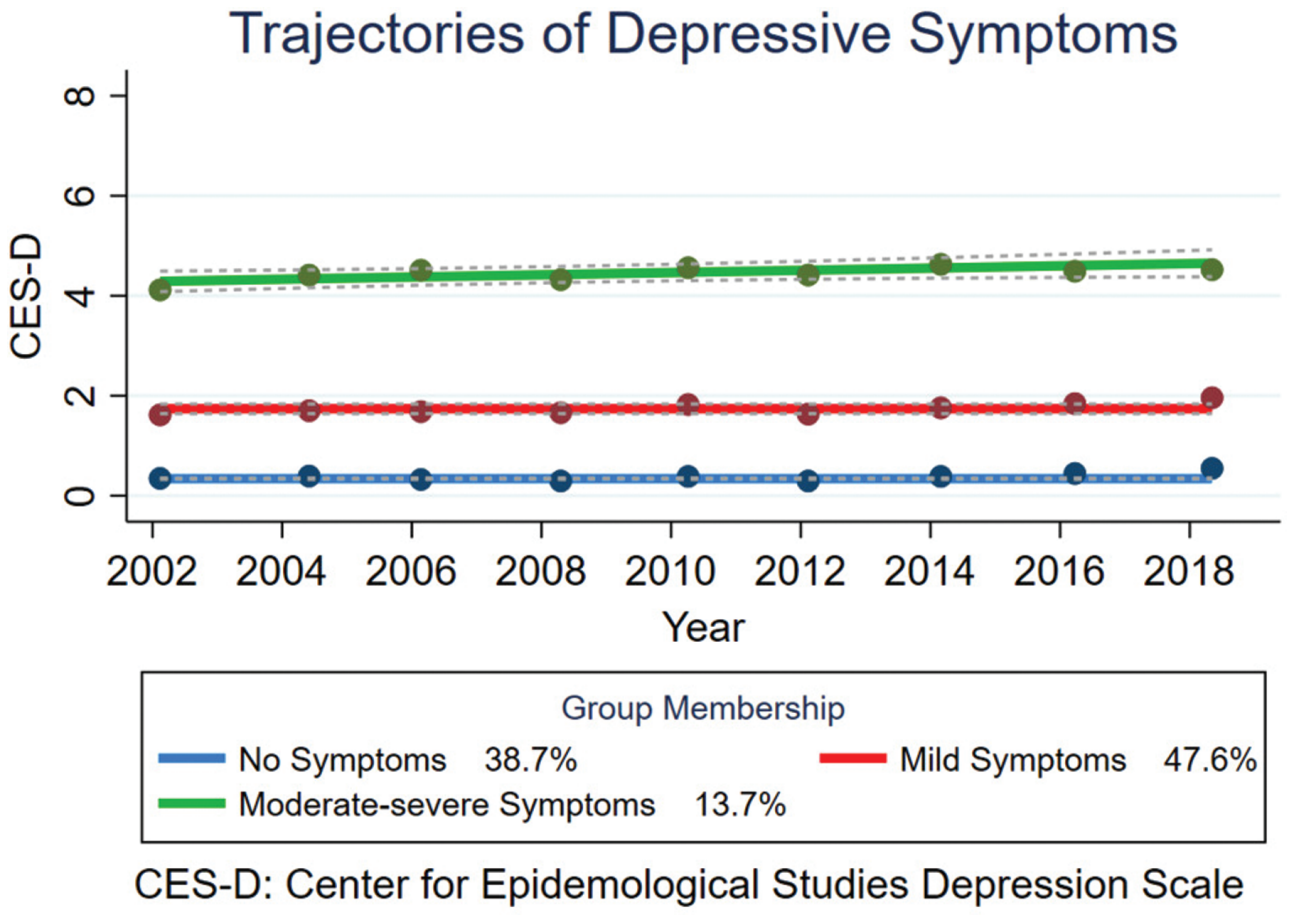
Trajectories of depressive symptoms among 7 050 adults over the age of 60 y.

A 3-group model with the best combination of selection indices was chosen, with the lowest BIC and highest entropy and posterior probability values considered together. Through this, the model with a linear polynomial in the order of 0, 0, 1 was chosen. Supplementary Tables S1–S3 provide detail on the 6 best-fit models including the final model selected, the posterior probabilities for the assignment of members for the selected model, and the summary of the selected model by each trajectory. Three trajectories were identified from the analysis: “no” (*n* = 2 726), “mild” (*n* = 3 357), and “moderate-severe” (*n* = 967) symptom trajectories (Figure 1). Depressive symptoms in all trajectories were broadly stable over time, with a weak trend toward increasing symptoms in the moderate-severe trajectory. The median CES-D scores were 0 (interquartile range [IQR]: 0.0–0.0), 1 (IQR: 1.0–2.0), and 4 (IQR: 3.0–6.0) for the no-, mild-, and moderate-severe symptom trajectories, respectively. The median CES-D score of the moderate-severe symptom trajectory met the threshold for depressive symptoms. The proportion of older adults with depressive symptoms (CES-D ≥ 4) at baseline in each trajectory were 0.4%, 12.4%, and 65.4% for no-, mild-, and moderate-severe symptoms, respectively (Supplementary Table S10). The characteristics of each trajectory are detailed in Supplementary Table S10.

#### Trajectories comparison

There were statistically significant differences between the 3 trajectories for the prevalence of depressive symptoms, age, sex, ethnicity, marital status, activities of daily living, instrumental activities of daily living, total number of comorbidities, total number of mobility limitations, number of times fallen, CASP-19 score, self-rated general health, life satisfaction score, if health limited ability to work, pain, physical activity level, having a longstanding illness, comorbidities of the musculoskeletal, respiratory, circulatory and metabolic systems, falls history, alcohol consumption, social network, smoking status, if they fractured their hip or had depression or manic depression (*p* < .001; Supplementary Table S12). There was no difference between the 3 trajectories for body mass index (BMI), diseases of the nervous system, neoplasms, mental disorders, or the management approach for depression (*p* > .01).

### Older Adults With Hip Fracture

**Figure 2.**
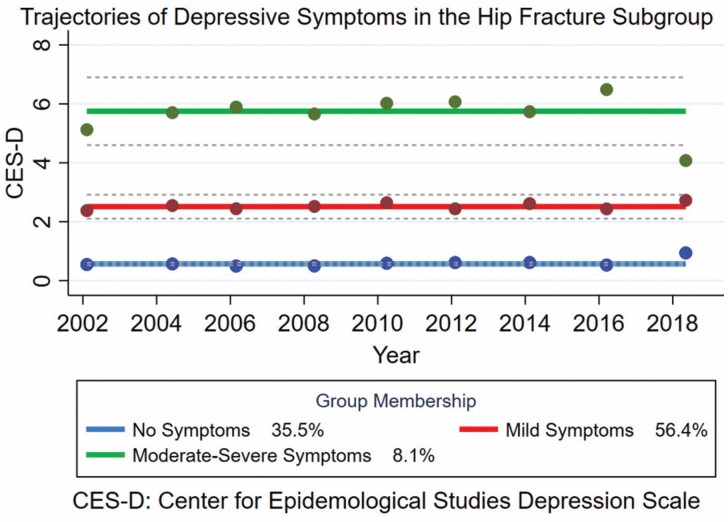
Trajectories of depressive symptoms among 384 adults over the age of 60 y with hip fracture.

In the overall population, the prevalence of depressive symptoms was 15%. This is similar to the prevalence after the removal of participants with a history of hip fracture (14.6%). In the overall population, there were 3.6%, 6.2%, and 8.2% of patients with hip fracture in the no-, mild-, and moderate-severe symptom trajectories, respectively. Overall, 384 (5.5%) patients in the ELSA data set suffered a hip fracture between 2002 and 2019. Of these, 87 (22.7%) patients experienced depressive symptoms at the fracture wave. A 3-group model in the order of 0, 0, 0 was the most appropriate, when the best values of the model selection criteria were considered together. The summaries of the model selection by model and by the groups in the chosen model as well as the posterior probabilities for the selected model are presented in Supplementary Tables S4–S6. Three trajectories were identified from the analysis: “no” (*n* = 138), “mild” (*n* = 219), and “moderate-severe” (*n* = 27) symptoms (Figure 2). Depressive symptoms in all trajectories were largely stable over time, with a significant positive linear slope in the moderate-severe symptom trajectory. The 95% confidence intervals (CI) in this trajectory are relatively wide, suggesting a range of scores between approximately 5 and 7, and some fluctuation in scores over time within this range. The median CES-D scores in the trajectories were 0 (IQR: 0.0–1.0), 2 (IQR: 1.0–5.0), and 6.5 (IQR: 6.0–7.5) for the no-, mild-, and moderate-severe symptom trajectories, respectively. The median CES-D score of the moderate-severe symptom trajectories met the threshold for depressive symptoms. In each trajectory, the proportion of older adults with depressive symptoms was 0.7%, 28.8%, and 85.2% in the no-, mild-, and moderate-severe symptom trajectories, respectively (Supplementary Table S11). The characteristics of each trajectory are detailed in Supplementary Table S11.

#### Trajectory comparisons

There were statistically significant differences between the 3 trajectories for prevalence of depressive symptoms, marital status, if health limited ability to work, self-rated general health, pain, total number of mobility limitations, having a longstanding illness, CASP-19 score and life satisfaction score (*p* < .001). There was no significant difference between the 3 trajectories for ethnicity, age, sex, BMI, activities of daily living, instrumental activities of daily living, total number of comorbidities, falls history, physical activity level, comorbidities of the circulatory, respiratory, musculoskeletal, and metabolic systems, mental disorders and neoplasms, alcohol consumption, smoking status, social networks, and whether they had diagnosis of depression or manic depression (*p* > .0001; Supplementary Table S13). No adult with hip fracture reported receiving medication or counseling (Supplementary Table S11).

#### Missing data analysis

In the main analysis, 40% of individuals had their CES-D scores recorded in at least 5 waves. Trajectories identified using data from the first 5 waves of data only, and by excluding missing depressive symptoms, data were comparable to the main analysis with good kappa agreement for group classification (Supplementary Table S14). For the overall sample, in both analyses, the trajectory shapes mirror the main analysis with a slightly more pronounced increase in symptoms in the moderate-severe symptom trajectory. In the hip fracture group, the analyses showed the trajectories with moderate-severe symptoms had less severe symptoms than the moderate-severe trajectory of the main analysis (median CES-D scores: <5 of 8 vs 6 of 8). In both samples, for the analysis excluding missing data, median CES-D scores for the trajectories did not meet the threshold for depressive symptoms (Supplementary Figures 1–4).

## Discussion

### Main Findings

The results of this study suggest 3 distinct trajectories of depressive symptoms in adults aged over 60 in England and in those with hip fracture. Trajectories followed a similar pattern in the overall and hip fracture populations; however, the distribution of depressive symptoms is shifted toward the moderate-severe group for those in the hip fracture subgroup. Individuals in the trajectory in which the median CES-D score met the threshold for depressive symptoms had different characteristics, for example, they were older, more likely to be female, less likely to be married or in a civil partnership and exhibited worse health and quality of life outcomes.

### Trajectories

All trajectories remained broadly stable across the study period, with slight uptrends in the trajectories in which the median CES-D score exceeded the threshold for depressive symptoms. A systematic review of depressive symptom trajectories across the life span identified 2 studies with 3 older adult trajectories—minimal, emerging (subclinical), and moderate or increasing and persistent (25–27). The results of the current study align with the classification of these 3-group trajectory studies. However, no major or consistent increase or decrease in symptoms was observed over time in the current study. The studies varied in terms of duration (10 years of follow-up (27) and 20 years of follow-up (25)), and geographical region (United States (27) and France (25)). These variations may be attributed to compositional differences such as gender, age, and racial demographics of populations which have previously been shown to influence the trajectories for depressive symptoms (26).

Among older adults after hip fracture, Cristancho et al. identified 3 trajectories “resilient,” “distressed,” and “depressed” (5). In contrast to the current study, they noted an uptrend in symptoms for those with depressive symptoms over time (5). Liu and colleagues identified 2 trajectories of depressive symptoms in the one-year following hip fracture, one trajectory experienced a decline in their symptoms and the other an overall uptrend in symptoms (28). The changes in trajectories over time noted by these earlier studies may relate to the duration and timing of follow-up. Both Cristancho and Liu studies followed participants for 1 year after hip fracture, which has been shown to be the most significant period for the risk of developing depressive symptoms after hip fracture (5,28). The 17-year period investigated by the current study potentially averaged out annual increases and decreases that may have occurred to give overall flat trajectories. These flat trajectories may indicate there is no “optimal” time to intervene, and it is never too late to support an older adult experiencing depressive symptoms with hip fracture. These results also suggest symptoms may persist in the longer term after initial recovery, highlighting a potential value for continual monitoring and awareness from health professionals.

A higher median CES-D score was observed for the moderate-severe trajectory for the hip fracture subgroup (6.5 [IQR: 6.0–7.5]) compared with the moderate-severe trajectory for the overall population (4.0 [IQR: 3.0–6.0]). This may suggest greater symptom severity among those with a history of hip fracture. Although the sample size for the moderate-severe symptom trajectory in the hip fracture group was small, the median CES-D score and its IQR were more compact. This suggests there was less variation in the population of the moderate-severe symptom trajectory among patients with hip fracture than the overall sample. This is not surprising, as the hip fracture group is expected to be more homogeneous than the overall, larger sample.

### Characteristics of Trajectories

There were differences in the characteristics of older adults within each trajectory, overall, and for those with hip fracture. For example, individuals in the trajectory with moderate-severe symptoms were older, more likely to be female, less likely to be married or in a civil partnership, and exhibited worse health and quality of life outcomes. These results are comparable to Musliner et al. who reported in the higher symptom trajectories, individuals were more likely to be female, were smokers, and have poor self-rated general health (26). For adults who sustain a hip fracture, those with depressive symptoms were less likely to be married or in a civil partnership, reported higher pain scores, and exhibited worse outcomes in certain health and quality of life factors than those without depressive symptoms. For all characteristics, these between trajectories comparisons were completed post hoc and should be confirmed with future appropriately powered prognostic factor analyses (29).

Similar to the study by Liang et al., the current study noted those alone exhibited higher depressive symptoms than those married both overall and for those with hip fracture (30). In contrast, Liu et al. noted no association between marital status and depressive symptoms among older adults after hip fracture (28). These differing findings are surprising given spousal bereavement is a known risk factor of depression (31) and the close relationship between loneliness and depressive symptoms (32). A potential explanation for these differences may relate to interactions between marital status and gender. Indeed, Montagnier et al. reported an association between marital status and persistent depression, only for women who were widowed (25).

The extent to which participants in previous studies were in receipt of pharmacological and/or nonpharmacological management for depressive symptoms over time is poorly described. The effectiveness of these management approaches in the general adult population is well established (33) and are likely to influence the observed trajectories. For the population with hip fracture, Li et al. (34) found psychological support therapy significantly decreased self-rated depression scores. Burns et al. (35) found marginal reductions in depression scores in hip fracture patients who received psychiatric intervention in the form of visits and phone calls with a psychiatric nurse. Several studies indicate promise (34–37), but the optimal approach is uncertain and limited by methodological concerns including underpowered results (35) and study interventions only being administered to those with either mild or severe depression only rather than a heterogeneous population regarding depression severity (35,37). Using a heterogeneous population better represents the population of people with depressive symptoms and therefore would provide more generalizable results for the wider population (38). Therefore, whether specific nonpharmacological interventions are warranted in this population are unknown. For the current study, individuals in the overall population were more likely to receive medication rather than counseling or both medication and counseling however, no significant differences were found between the management approach and trajectory membership. None of the older adults with hip fracture reported receiving medication or counseling. Therefore, replication of the research investigating the interactions between management approaches within trajectories for depression overall and for those with hip fracture is warranted.

### Limitations

There are limitations to this study. First, the ELSA sample comprises predominantly White community-dwelling individuals limiting generalizability of results to non-White and residential/nursing care populations. Second, data were missing for several variables that were not collected across all waves. We assessed the sensitivity of our findings to missingness by conducting the analyses using data from the first 5 waves only and then by excluding missing depressive symptoms. We found the results comparable to the main analysis. Third, there is potential selection bias as the year individuals suffered their hip fracture was not available and may relate to the trajectories of depressive symptoms. This also meant we were unable to see the level of change in depressive symptoms after hip fracture. Fourth, there are no gold standards for sample size for latent class analyses. Sample sizes as small as 30 have been shown to produce valid results for simple latent class modeling with distinct classes, whereas sample sizes from 300 to 1 000 have been suggested for models with multiple indicators and classes. The main concerns regarding inadequate sample size are unpowered models which cannot detect classes, inaccurate solutions produced, and small but relevant classes being missed (39). However, the high posterior probabilities, entropy, and the meaningful components of the different trajectories for our models suggest adequate classifications. Finally, we may have overestimated the prevalence of depressive symptoms by using the CES-D compared with diagnostic criteria (40,41).

## Conclusions

Older adults, and those with hip fracture, follow one of the 3 trajectories of depressive symptoms that are broadly stable over time. This may suggest it is never too late to target depressive symptoms for these patients as symptoms may persist in the longer term. Only one of the 3 trajectories had a median CES-D score, which met the threshold for depressive symptoms. Depressive symptoms’ prevalence was higher for those with hip fracture and the distribution of depressive symptoms consistently over time is shifted toward the moderate-severe group for those in the hip fracture subgroup when compared with the overall population. Results suggest a role of factors including age, gender, and marital status in depressive symptom trajectories both overall and for those with hip fracture which should be explored in future research.

## Supplementary Material

glac182_suppl_Supplementary_Material
