# Supplementary material for: Trajectories of Depressive Symptoms Among Older Adults and in Adults With Hip Fracture: Analysis From the English Longitudinal Study of Ageing
Source: J Gerontol A Biol Sci Med Sci. 2022 Sep 8;77(12):2453–8. doi: 10.1093/gerona/glac182 (PMC9799186; doi:10.1093/gerona/glac182)
Supplement: glac182_suppl_Supplementary_Material [file glac182_suppl_supplementary_material.pdf]

Number of Tables: 14

Number of Figures: 4

Elements:

eTable 1 Summary of model selection criteria – By Model

eTable 2 Posterior probabilities for the assignment of members to different trajectories

eTable 3 Summary of model selection – By Group for the selected model

eTable 4 Summary of model selection – By Model

eTable 5 Posterior probabilities for the assignment of members to different trajectories

eTable 6 Summary of model selection – By Group for the selected model

eTable 7 Summary and Patterns of Missing Data in the Overall Population

eTable 8 Summary and Patterns of Missing Data in the Hip Fracture Population

eTable 9 Characteristics of 7,050 patients overall and by the presence of depressive symptoms at baseline (score of ≥4 on CES-D score)

eTable 10 Prevalence of depressive symptoms and characteristics of each trajectory group for the total population

eTable 11 Prevalence of depressive symptoms and characteristics of each trajectory group for the hip fracture population

eFigure 1 Trajectory models in overall population using the first five waves of Depressive Symptoms data

eFigure 2 Trajectory models in overall population using complete cases of Depressive Symptoms data

eFigure 3 Trajectory models in the hip fracture population using the first five waves of Depressive Symptoms data

eFigure 4 Trajectory models in the hip fracture population using complete cases of Depressive Symptoms data

eTable 12 Chi-squared Test Results – Overall Population

eTable 13 Chi-squared Test Results – Hip Fracture Population

eTable 14 Kappa Agreements for Sensitivity Analysis for Missing Data in Trajectory Models

Model Selections – Overall Population

eTable 1 Summary of model selection criteria – By Model

Number of groups	Parameters by group*	BIC	Entropy
3	1 1 1	-60955.72	0.721
3	0 1 1	-60972.96	0.714
3	0 0 1	-60992.90	0.715
4	1 1 1 1	-60633.36	0.681
4	1 1 1 0	-60630.48	0.682
4	1 1 0 0	-60640.01	0.685

Polynomial shapes: 0=intercept, 1=linear, 2=quadratic, 3=cubic
BIC: Bayesian information criterion (for the total number of participants)

eTable 2 Posterior probabilities for the assignment of members to different trajectories

Probability	Group 1<br>n=2726	Group 2<br>n=3357	Group 3<br>N=967
Group 1	0.873 (0.148)	0.127 (0.147)	0.000 (0.001)
Group 2	0.088 (0.129)	0.855 (0.141)	0.057 (0.107)
Group 3	0.000 (0.001)	0.122 (0.145)	0.878 (0.146)

Mean (SD)

eTable 3 Summary of model selection – By Group for the selected model

Group	Group Membership (%)	Parameter	Estimate (SE)	Probability
1	38.7*	Intercept	-1.278 (0.0449)	<0.001
2	47.6*	Intercept	1.440 (0.0408)	<0.001
3	13.7*	Intercept	-43.26 (15.257)	0.005
		Slope - Linear	0.0238 (0.008)	0.002

*<0.001

SE: Standard Error

Model Selections – Hip Fracture Population

eTable 4 Summary of model selection – By Model

Number of groups	Parameters by group*	BIC	Entropy
3	0 0 0	-3843.02	0.782
3	1 1 1	-3848.65	0.777
3	1 1 2	-3851.63	0.777
3	2 1 0	-3850.03	0.788
4	1 1 0 0	-3826.04	0.704
4	1 1 0 1	-3828.27	0.7

Polynomial shapes: 0=intercept, 1=linear, 2=quadratic, 3=cubic
BIC: Bayesian information criterion (for the total number of participants)

eTable 5 Posterior probabilities for the assignment of members to different trajectories

Probability	Group 1<br>n=138	Group 2<br>n=219	Group 3<br>n=27
Group 1	0.895 (0.146)	0.105 (0.146)	0.000 (0.000)
Group 2	0.059 (0.106)	0.907 (0.119)	0.034 (0.080)
Group 3	0.000 (0.000)	0.121 (0.159)	0.879 (0.159)

eTable 6 Summary of model selection – By Group for the selected model

Group	Group Membership (%)	Parameter	Estimate (SE)	Probability
1	35.5*	Intercept	-0.711 (0.167)	<0.001
2	56.7*	Intercept	2.355 (0.147)	<0.001
3	8.1*	Intercept	5.958 (0.433)	<0.001

*p<0.001
SE: Standard Error

eTable 7 Summary and Patterns of Missing Data in the Overall Population

No. of waves with missing data	Frequency (%) *	Wave(s) with missing data	Frequency (%) †
1	540 (7.66)	Wave 9	328 (60.74)
2	483 (6.85)	Waves 8-9	329 (68.12)
3	1100 (15.6)	Waves 1-3, 7-9	961 (87.36)
4	719 (10.2)	Waves 6-9	355 (49.37)
5	795 (11.28)	Waves 5-9	468 (58.87)
6	981 (13.91)	Waves 4-9	656 (66.87)
7	1225 (17.38)	Waves 3-9	886 (72.33)

*Percentage of people with missing data for 1-7 waves
†Percentage of people with missing data at the corresponding wave(s) in relation to the total number of people with missing data

eTable 8 Summary and Patterns of Missing Data in the Hip Fracture Population

No. of waves with missing data	Frequency (%)	Wave(s) with missing data	Frequency (%) *
1	37 (9.64)	Wave 9	26 (70.27)
2	39 (10.16)	Waves 8-9	25 (64.1)
3	69 (17.97)	Waves 7-9	36 (52.17)
4	31 (8.07)	Waves 6-9	18 (58.06)
5	47 (12.24)	Waves 5-9	31 (65.96)
6	43 (11.2)	Waves 4-9	37 (86.05)
7	57 (14.84)	Waves 3-9	38 (66.67)

*Percentage of people with missing data for 1-7 waves
†Percentage of people with missing data at the corresponding wave(s) in relation to the total number of people with missing data

eTable 9 Characteristics of 7,050 patients overall and by the presence of depressive symptoms at baseline (score of ≥4 on CES-D score)

	Total	No depressive symptoms [a]	Depressive symptoms [a]
	N=7,050	N=5,991	N=1,059
Age			
60-69	3,846 (54.6)	3,318 (55.4)	528 (49.9) *
70-79	2,428 (34.4)	2,049 (34.2)	379 (35.8) *
80-89	739 (10.5)	594 (9.9)	145 (13.7) *
90-99	37 (0.5)	30 (0.5)	7 (0.7) *
BMI			
<18.5	65 (1.1)	52 (1.0)	13 (1.6) *
18.5-24.9	1,576 (26.6)	1,363 (26.8)	213 (25.7) *
25-29.9	2,572 (43.4)	2,251 (44.2)	321 (38.8) *
30+	1,708 (28.8)	1,427 (28.0)	281 (33.9) *
Hip Fracture	384 (5.4)	297 (5.0)	87 (8.2) *
Pain	2,838 (40.3)	2,148 (35.9)	690 (65.2) *
Sex			
Male	3,257 (46.2)	2,903 (48.5)	354 (33.4) *
Female	3,793 (53.8)	3,088 (51.5)	705 (66.6) *
Ethnicity			
White	6,889 (97.7)	5,874 (98.0)	1,015 (95.8) *
Non-White	161 (2.3)	117 (2.0)	44 (4.2) *
Marital Status			
Single	337 (4.8)	282 (4.7)	55 (5.2) *
Married or in Civil Partnership	4,586 (65.0)	4,061 (67.8)	525 (49.6) *
Separated or Divorced	606 (8.6)	485 (8.1)	121 (11.4) *
Widowed	1,521 (21.6)	1,163 (19.4)	358 (33.8) *
Health			
Health limited ability to work	2,609 (37.0)	1,962 (32.7)	647 (61.1) *
Self-rated general health			
Excellent	809 (11.5)	783 (13.1)	26 (2.5) *
Very Good	2,016 (28.6)	1,857 (31.0)	159 (15.0) *
Good	2,336 (33.1)	2,054 (34.3)	282 (26.6) *
Fair	1,397 (19.8)	1,039 (17.3)	358 (33.8) *
Poor	492 (7.0)	258 (4.3)	234 (22.1) *
Mobility			
Total Mobility Limitations			
0-2	6,105 (86.6)	5,338 (89.1)	767 (72.5) *
3-5	945 (13.3)	653 (10.9)	292 (27.5) *
Total ADL's find Difficult			
0-3	6,088 (97.1)	5,252 (98)	836 (91.8) *
4-6	184 (2.9)	109 (2)	75 (8.2) *
Total IADL's find Difficult			
0-4	6,149 (98.1)	5,282 (98.5)	867 (95.2) *
5-8	123 (1.9)	79 (1.4)	44 (4.8) *
Comorbidities			
Circulatory system diseases	461 (8.1)	372 (7.8)	89 (9.7)
Respiratory system diseases	604 (10.2)	462 (9.3)	142 (14.9) *
Nervous system diseases	12 (0.2)	9 (0.2)	3 (0.3)
MSK diseases	1,834 (28.3)	1,451 (26.5)	383 (37.8) *

	Total	No depressive symptoms [a]	Depressive symptoms [a]
Metabolic diseases	438 (6.2)	342 (5.7)	96 (9.1) *
Mental disorders	21 (0.3)	15 (0.3)	6 (0.6)
Neoplasms	154 (2.2)	131 (2.2)	23 (2.2)
Total Comorbidities			
0-2	6,997 (99.3)	5,960 (99.4)	1,037 (98) *
3-4	53 (0.7)	31 (0.5)	22 (2.0) *
Falls			
Had a fall	2,085 (29.6)	1,599 (26.7)	486 (45.9) *
Number of times fallen			
1-5	4,671 (94.4)	3,938 (95.3)	733 (89.3) *
6-10	196 (4)	140 (3.3)	56 (6.8) *
11-30	61 (0.9)	38 (0.7)	23 (2.8) *
Smoking Status			
Current smoker	761 (14.1)	593 (13.0)	168 (19.9) *
Stopped smoking	4,637 (85.9)	3,961 (87.0)	676 (80.1) *
Alcohol Consumption			
More than twice a day, daily or almost daily	1,845 (26.2)	1,634 (27.3)	211 (20.0) *
3-6 times/week	305 (4.3)	280 (4.7)	25 (2.4) *
1-2 times/week	1,892 (26.9)	1,657 (27.7)	235 (22.3) *
1-2 times/month	728 (10.3)	628 (10.5)	100 (9.5) *
Once every couple months	103 (1.5)	85 (1.4)	18 (1.7) *
1-2 times/year	1,276 (18.1)	1,017 (17.0)	259 (24.5) *
None in the last 12 months	885 (12.6)	678 (11.3)	207 (19.6) *
Physical Activity			
Sedentary	588 (9.6)	416 (7.9)	172 (19.1) *
Low	1,749 (28.4)	1,386 (26.4)	363 (40.3) *
Moderate	2,934 (47.7)	2,636 (50.2)	298 (33.1) *
High	883 (14.3)	815 (15.5)	68 (7.5) *
Social Network			
Member of an organisation	2,115 (30.7)	1,680 (28.5)	435 (42.9) *
Quality of Life			
Limiting long-standing illness	3,478 (56.0)	2,715 (52.2)	763 (76.3) *
Life Satisfaction [b]			
Strongly agree	763 (13.1)	715 (14.2)	48 (6.1) *
Agree	2,999 (51.4)	2,765 (54.8)	234 (29.7) *
Slightly agree	862 (14.8)	707 (14.0)	155 (19.6) *
Neither agree nor disagree	490 (8.4)	391 (7.8)	99 (12.5) *
Slightly disagree	372 (6.4)	277 (5.5)	95 (12.0) *
Disagree	236 (4.0)	131 (2.6)	105 (13.3) *
Strongly disagree	109 (1.9)	56 (1.1)	53 (6.7) *
CASP-19 Total Score			
0-29	520 (7.7)	317 (5.5)	203 (20.8) *
30-57	6,255 (92.3)	5,480 (94.5)	775 (79.2) *
Depression			
Has depression/manic depression	153 (2.2)	94 (1.6)	59 (5.6) *

	Total	No depressive symptoms [a]	Depressive symptoms [a]
Medication or counselling			
Medication	83 (38.4)	57 (39.9)	26 (35.6)
Counselling	10 (4.6)	7 (4.9)	3 (4.1)
Both medication and counselling	45 (20.8)	30 (21.0)	15 (20.5)
None	78 (36.1)	49 (34.3)	29 (39.7)

Note: Data are presented as n (%) for categorical measures.
[a] No Depressive Symptoms = CES-D Score <4, Depressive Symptoms = CES-D Score ≥ 4
[b] Life Satisfaction Question: Is participant satisfied with their life
BMI: Body Mass Index; ADL: Activities of Daily Living; IADL: Instrumental Activities of Daily Living; CASP-19 – CASP-19 Quality of Life Scale
* p<0.005

eTable 10 Prevalence of depressive symptoms and characteristics of each trajectory group for the total population

	No symptoms (n=2726)		Mild Symptoms (n=3357)		Moderate-severe Symptoms (n=967)	
	N	%	N	%	N	%
Prevalence of Depressive Symptoms [a]*						
CES-D<4	2715	99.6	2941	87.6	335	34.6
CES-D≥4	11	0.4	416	12.39	632	65.36
CES-D Total Score [a b]	0.0	0.0-0.0	1.0	1.0-2.0	4.0	3.0-6.0
Age*						
60-69	1711	62.8	1697	50.6	438	45.3
70-79	831	30.5	1226	36.5	371	38.4
80-89	174	6.4	411	12.2	154	15.9
90-99	10	0.4	23	0.7	4	0.4
BMI [c]						
<18.5	21	0.9	31	1.1	13	1.7
18.5-24.9	685	28.8	701	25.0	190	25.5
25-29.9	1099	46.2	1192	42.6	281	37.7
30+	572	24.1	875	31.3	261	35.0
Hip Fracture*	97	3.6	208	6.2	79	8.2
Pain*	631	23.1	1557	46.4	650	67.2
Sex*						
Male	1554	57.0	1404	41.8	299	30.9
Female	1172	43.0	1953	58.2	668	69.1
Ethnicity*						
White	2677	98.2	3288	97.9	924	95.6
Non-White	49	1.8	69	2.1	43	4.4
Marital Status*						
Single	127	4.7	160	4.8	50	5.2
Married or in Civil Partnership	2005	73.6	2094	62.4	487	50.4
Separated or Divorced	208	7.6	277	8.3	121	12.5
Widowed	386	14.2	826	24.6	309	32
Health						
Health limited ability to work*	529	19.4	1438	42.8	642	66.4
Self-rated general health*						
Excellent	526	19.3	261	7.8	22	2.3
Very Good	1053	38.6	842	25.1	121	12.5
Good	848	31.1	1229	36.6	259	26.8
Fair	271	9.9	780	23.2	346	35.8
Poor	28	1.0	245	7.3	219	22.6
Mobility*						
Total Mobility Limitations						
0-2	2,640	96.9	2,819	84.1	646	66.8
3-5	86	3.2	538	16	321	33.1
Total ADL's find Difficult						
0-3	2,433	99.5	2,890	97	765	90.3
4-6	11	0.4	90	3	83	9.8
Total IADL's find Difficult						
0-4	2,427	99.3	2,919	97.9	803	94.7
5-8	17	0.7	61	2	45	5.3
Comorbidities						

	No symptoms (n=2726)		Mild Symptoms (n=3357)		Moderate-severe Symptoms (n=967)	
Circulatory system diseases* [c]	126	6.1	250	9.1	85	10.1
Respiratory system diseases* [c]	165	7.7	310	10.7	129	14.8
Nervous system diseases [c]	2	0.1	7	0.3	3	0.4
MSK diseases* [c]	436	18.3	1005	31.9	393	41.7
Metabolic diseases*	129	4.7	218	6.5	91	9.4
Mental disorders	4	0.1	10	0.3	7	0.7
Neoplasms	49	1.8	81	2.4	24	2.5
Total Comorbidities*						
0-2	2719	99.8	3328	99.1	950	98.3
3-4	7	0.3	29	0.8	17	1.8
Falls						
Had a fall*	572	21.0	1075	32	438	45.3
Number of times fallen* [c]						
1-5	1630	97.6	2338	93.8	703	89.2
6-10	34	2.1	106	4.3	56	7.1
11-30	4	0.3	35	1.2	22	2.8
Smoking Status* [c]						
Current smoker	224	11.2	362	13.8	175	22.3
Stopped smoking	1773	88.8	2256	86.2	608	77.7
Alcohol Consumption* [c]						
More than twice a day, daily or almost daily	822	30.2	829	24.8	194	20.1
3-6 times/week	164	6.0	120	3.6	21	2.2
1-2 times/week	805	29.6	882	26.4	205	21.2
1-2 times/month	279	10.2	361	10.8	88	9.1
Once every couple months	31	1.1	55	1.6	17	1.8
1-2 times/year	400	14.7	652	19.5	224	23.2
None in the last 12 months	221	8.1	448	13.4	216	22.4
Physical Activity* [c]						
Sedentary	82	3.4	325	11.1	181	21.5
Low	482	20.1	903	30.9	364	43.3
Moderate	1341	56.0	1343	46	250	29.8
High	491	20.5	347	11.9	45	5.4
Social Network [c]						
Member of an organisation*	648	24.1	1063	32.4	404	43.7
Quality of Life						
Limiting long-standing illness* [c]	898	40.9	1850	60.1	730	78.2
Life Satisfaction* [c]						
Strongly agree	417	17.8	309	11.2	37	5.1
Agree	1508	64.5	1318	47.7	173	23.6
Slightly agree	240	10.3	499	18.1	123	16.8
Neither agree nor disagree	97	4.1	284	10.3	109	14.9
Slightly disagree	50	2.1	206	7.5	116	15.8
Disagree	23	1.0	106	3.8	107	14.6
Strongly disagree	3	0.1	39	1.4	67	9.2
CASP-19 Total Score* [c]						
0-29	83	3.1	235	7.3	202	22.7
30-57	2598	96.9	2969	92.7	688	77.3
Depression						
Has depression/manic depression* [c]	19	0.7	66	2	68	7

	No symptoms (n=2726)		Mild Symptoms (n=3357)		Moderate-severe Symptoms (n=967)	
Medication or counselling						
Medication	13	39.4	39	37.1	31	39.7
Counselling	0	0.0	4	3.8	6	7.7
Both medication and counselling	7	21.2	23	21.9	15	19.2
None	13	39.4	39	37.1	26	33.3

Note: [a] Depressive Symptoms: Prevalence of Depressive Symptoms per trajectory group; CES-D Total Score

[b] Median (Interquartile Range (IQR))

[c] Missing Values: BMI: n=1129; ADL: n=778; IADL: n=778; CASP-19: n=275; Limiting long-standing illness: n=844; Circulatory system diseases: n=1389; Respiratory system diseases: n=1144; Nervous system diseases: n=1392; MSK diseases: n=571; number of times fallen: n=2101; smoking: n=1652; alcohol consumption: n=16; physical activity: n=896; member of an organisation: n=150; number of close family members: n=519; life satisfaction: n=1219; (manic) depression: n=6834

* Chi-squared p<0.001

eTable 11 Prevalence of depressive symptoms and characteristics of each trajectory group for the hip fracture population

	No symptoms (n=138)		Mild Symptoms (n=219)		Severe Symptoms (n=27)		Total (n=384)	
	N	%	N	%	N	%	N	%
Prevalence of Depressive Symptoms [a] *								
CES-D<4	137	99.3	156	71.2	4	14.8	297	77.3
CES-D≥4	1	0.7	63	28.8	23	85.2	87	22.7
CES-D Total Score [a b]	0	0.0-1.0	2	1.0-5.0	6.5	6.0-7.5	2.0	0.0-4.0
Age [d]	79	74-84	79	72-84	78	73-83	79	73-84
60-69	13	10.4	41	20.3	1	4.5	55	15.8
70-79	50	40.0	65	32.2	12	54.5	127	36.4
80-89	60	48.0	87	43.1	9	40.9	156	44.7
90-99	2	1.6	9	4.5	0	0.0	11	3.2
BMI [d]								
18.5-24.9	10	41.7	12	32.4	3	60.0	25	37.9
25-29.9	11	45.8	13	35.2	1	20.0	25	37.9
30+	3	12.5	12	32.4	1	20.0	16	24.2
Pain* [d]	44	34.9	126	63.3	18	75.0	188	53.9
Sex								
Male	55	39.9	71	32.4	3	11.1	129	33.6
Female	83	60.1	148	67.6	24	88.9	255	66.4
Ethnicity* [d]								
White	122	100.0	187	98.4	21	91.3	330	98.5
Non-White	0	0.0	3	1.6	2	8.7	5	1.5
Marital Status*								
Single	7	5.1	13	5.9	1	3.7	21	5.5
Married or in Civil Partnership	77	55.8	84	38.4	5	18.5	166	43.2
Separated or Divorced	5	3.6	19	8.7	7	25.9	31	8.1
Widowed	49	35.5	103	47.0	14	51.9	166	43.2
Health* [d]								
Health limited ability to work	45	41.7	116	74.4	16	88.9	177	62.8
Self-rated general health								
Excellent	10	9.3	2	1.3	0	0.0	12	4.3
Very Good	30	27.8	20	12.8	1	5.6	51	18.1
Good	42	38.9	49	31.4	3	16.7	94	33.3
Fair	22	20.4	53	34.0	4	22.2	79	28.0
Poor	4	3.7	32	20.5	10	55.6	46	16.3
Mobility								
Total Mobility Limitations								
0-2	73	65.2	49	33.1	1	4.8	123	43.7
3-5	39	34.9	99	66.8	20	95.2	158	56.3
Total ADL's find Difficult								
0-3	101	90.1	113	76.8	14	66.6	228	81.4
4-6	11	9.9	34	23.1	7	33.3	52	18.6
Total IADL's find Difficult								
0-4	96	85.8	123	83.6	17	80.9	236	84.3
5-9	16	14.4	24	16.3	4	19	44	15.7
Comorbidities [d]								

	No symptoms (n=138)		Mild Symptoms (n=219)		Severe Symptoms (n=27)		Total (n=384)	
Circulatory system diseases	4	30.8	9	47.4	4	80.0	17	45.9
Respiratory system diseases	7	63.6	22	81.5	4	66.7	33	75.0
Nervous system diseases	0	0	0	0	0	0	0	0
MSK diseases	31	67.4	60	81.1	13	86.7	104	77.0
Metabolic diseases	8	7.1	9	6.1	2	9.5	19	6.8
Mental disorders	2	1.8	1	0.7	0	0.0	3	1.1
Neoplasms	8	7.1	6	4.1	0	0.0	14	5.0
Total Comorbidities								
0-2	111	99	145	97.9	20	95.3	276	98.3
3-4	1	0.9	3	2.1	1	4.8	5	1.8
Falls [d]								
Had a fall	75	59.5	143	71.9	20	83.3	238	68.2
Number of times fallen								
1-5	78	94	139	94.6	17	89.4	234	93.9
6-10	3	3.6	7	4.8	2	10.5	12	4.8
11-30	2	2.4	1	0.7	0	0	3	1.2
Smoking Status [d]								
Current smoker	3	11.5	16	27.6	2	20.0	21	22.3
Stopped smoking	23	88.5	42	72.4	8	80.0	73	77.7
Alcohol Consumption [d]								
More than twice a day, daily or almost daily	24	21.4	30	18.9	4	20.0	58	19.9
3-6 times/week	8	7.1	5	3.1	1	5.0	14	4.8
1-2 times/week	28	25.0	33	20.8	3	15.0	64	22.0
1-2 times/month	13	11.6	13	8.2	1	5.0	27	9.3
Once every couple months	7	6.3	8	5.0	0	0.0	15	5.2
1-2 times/year	12	10.7	28	17.6	4	20.0	44	15.1
None in the last 12 months	20	17.9	42	26.4	7	35.0	69	23.7
Physical Activity [d]								
Sedentary	6	18.2	36	45.0	4	44.4	46	37.7
Low	14	42.4	26	32.5	5	55.6	45	36.9
Moderate	8	24.2	15	18.8	0	0.0	23	18.9
High	5	15.2	3	3.8	0	0.0	8	6.6
Social Network [d]								
Member of an organisation	25	23.6	56	39.2	8	47.1	89	33.5
Quality of Life								
Limiting long-standing illness* [d]	69	71.1	164	87.2	24	96.0	257	82.9
Life Satisfaction* [d]								
Strongly agree	16	18.2	8	8.9	0	0.0	24	12.7
Agree	38	43.2	29	32.2	1	9.1	68	36.0
Slightly agree	18	20.5	16	17.8	1	9.1	35	18.5
Neither agree nor disagree	7	8.0	18	20.0	1	9.1	26	13.8
Slightly disagree	7	8.0	6	6.7	1	9.1	14	7.4
Disagree	2	2.3	10	11.1	4	36.4	16	8.5
Strongly disagree	0	0.0	3	3.3	3	27.3	6	3.2
CASP-19 Total Score* [d]								
0-29	4	4.0	24	17.6	4	28.6	32	12.7
30-57	97	96.0	112	82.4	10	71.4	219	87.3
Depression* [c] [d]								
Has depression/manic depression	2	1.8	3	2.0	2	9.5	7	2.5

Note: [a] Depressive Symptoms: The prevalence of Depressive Symptoms per trajectory group; CES-D Total Score
[b] Median (IQR)
[c] § No observations of medication or counselling data in those with hip fracture
[d] Missing Values: age: n=35; BMI: n=318; Mobility: n=103; ADL: n=104; IADL: n=104; CASP-19: n=133; ethnicity: n=49; Health limited ability to work: n=102; Self-rated general health: n=102; Limiting long-standing illness: n=74; Circulatory system diseases: n=347; Respiratory system diseases: n=340; Nervous system diseases: n=377; MSK diseases: n=249; metabolic diseases: n=103; mental disorders: n=103; neoplasms: n=103; Total Comorbidities: n=103; Had a fall: n=35; number of times fallen: n=135; overall pain: n=35; smoking: n=290; alcohol consumption: n=93; physical activity: n=262; member of an organisation: n=118; number of close family members: n=171; life satisfaction: n=195; (manic) depression: n=103
* Chi-squared p<0.001

eFigure 1 Trajectory models in overall population using the first five waves of Depressive Symptoms data

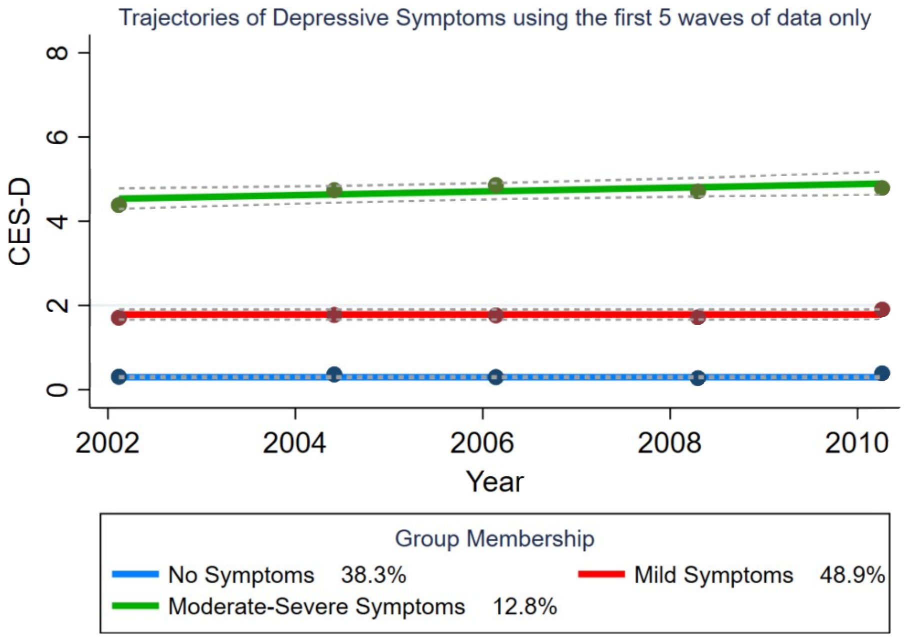

eFigure 2 Trajectory models in overall population using complete cases of Depressive Symptoms data

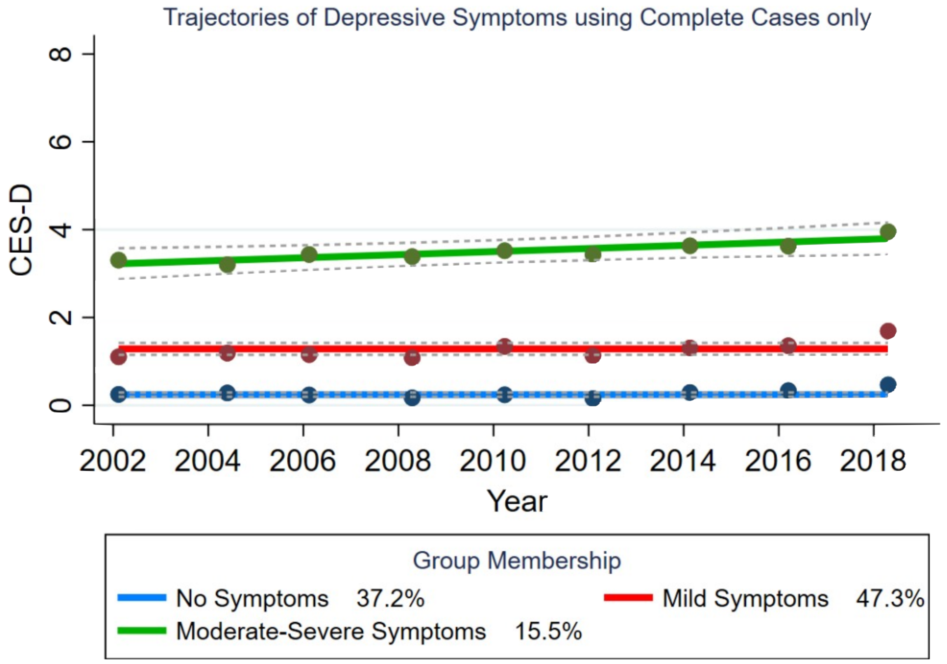

eFigure 3 Trajectory models in the hip fracture population using the first five waves of Depressive Symptoms data

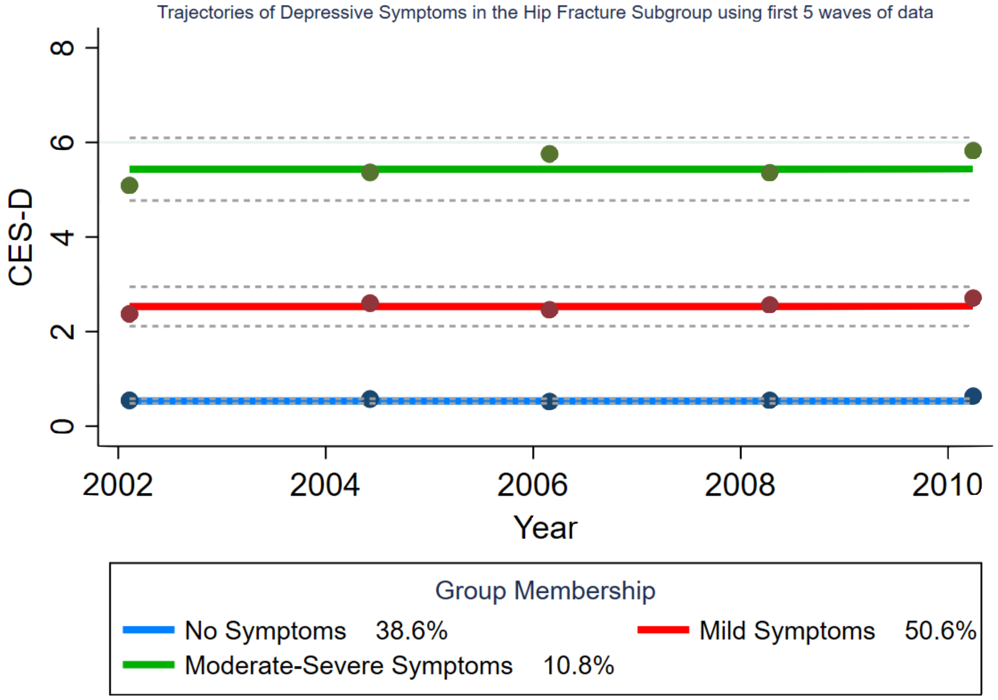

eFigure 4 Trajectory models in the hip fracture population using complete cases of Depressive Symptoms data

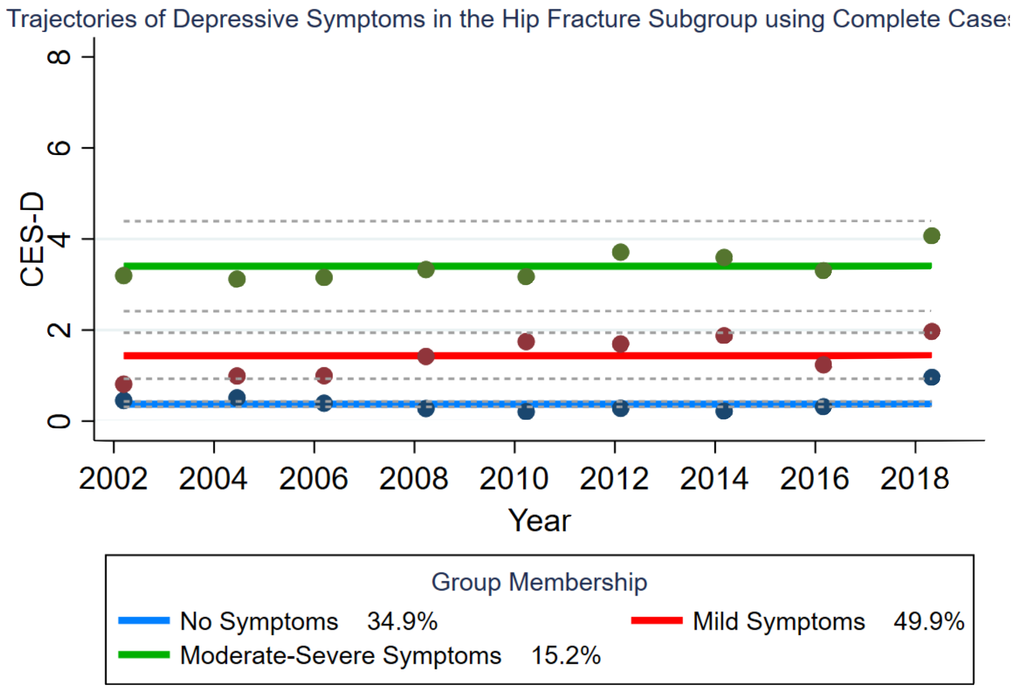

Bonferroni adjustment: alpha level/number of tests 0.05/33= 0.0015

eTable 12 Chi-squared Test Results – Overall Population

Characteristic	n	Chi-square	p-value
Depressive Symptoms		2393.87	<0.001
CESD<4	5991		
CESD>4	1059		
Self-rated general health		1239.33	<0.001
Excellent	809		
Very Good	2,016		
Good	2,336		
Fair	1,397		
Poor	492		
Life Satisfaction		1111.23	<0.001
Strongly agree	763		
Agree	2,999		
Slightly agree	862		
Neither agree nor disagree	490		
Slightly disagree	372		
Disagree	236		
Strongly disagree	109		
Total IADL's find Difficult		850.70	<0.001
0-4	6,149		
5-8	123		
Total Mobility Limitations		795.16	<0.001
0-2	6,105		
3-5	945		
Health limited ability to work	2,609	769.35	<0.001
Total ADL's find Difficult		683.73	<0.001
0-3	6,088		
4-6	184		
Pain	2,838	676.41	<0.001
Physical Activity		574.22	<0.001
Sedentary	588		
Low	1,749		
Moderate	2,934		
High	883		
Limiting long-standing illness	3,478	412.05	<0.001
CASP-19 Total Score		363.26	<0.001
0-29	520		
30-57	6255		
Total Comorbidities		298.10	<0.001
0-2	6,997		
3-4	53		
Number of times fallen		289.26	<0.001
1-5	4,671		

6-10	196		
11-30	61		
Sex		244.79	<0.001
Male	3,257		
Female	3,793		
Alcohol Consumption		243.24	<0.001
More than twice a day, daily or almost daily	1,845		
3-6 times/week	305		
1-2 times/week	1,892		
1-2 times/month	728		
Once every couple months	103		
1-2 times/year	1,276		
None in the last 12 months	885		
Diseases of the MSK System	1,834	221.18	<0.001
Had a fall	2,085	221.00	<0.001
Marital Status		219.17	<0.001
Single	337		
Married or in Civil Partnership	4,586		
Separated or Divorced	606		
Widowed	1,521		
Age		163.82	<0.001
60-69	3846		
70-79	2428		
80-89	739		
90-99	37		
Depression/manic depression	153	136.19	<0.001
Member of an organisation	2,115	133.26	<0.001
Smoking Status		57.87	<0.001
Current smoker	761		
Stopped smoking	4,637		
BMI		55.86	<0.001
<18.5	65		
18.5-24.9	1576		
25-29.9	2572		
30+	1708		
Hip Fracture	384	36.46	<0.001
Diseases of the respiratory system	604	35.61	<0.001
Metabolic diseases	438	27.68	<0.001
Ethnicity		23.95	<0.001
White	6,889		
Non-White	161		
Diseases of the circulatory system	461	18.65	<0.001
Mental disorders	21	8.01	0.02
Neoplasms	154	3.13	0.21
Diseases of the nervous system	12	2.35	0.31

Medication or counselling in last 2 years		3.80	0.7
Medication	83		
Counselling	10		
Both medication and counselling	45		
None	78		

eTable 13 Chi-squared Test Results – Hip Fracture Population

Characteristic	n	Chi-square	p-value
Depressive Symptoms		102.79	<0.001
CESD<4	297		
CESD>4	87		
Self-rated general health		56.25	<0.001
Excellent	12		
Very Good	51		
Good	94		
Fair	79		
Poor	46		
Life Satisfaction		51.86	<0.001
Strongly agree	24		
Agree	68		
Slightly agree	35		
Neither agree nor disagree	26		
Slightly disagree	14		
Disagree	16		
Strongly disagree	6		
Total Mobility Limitations		49.71	<0.001
0-2	123		
3-5	158		
Total IADL's find Difficult		39.38	0.003
0-4	236		
5-8	44		
Health limited ability to work	177	34.80	<0.001
Pain	188	29.66	<0.001
Marital Status		27.58	<0.001
Single	21		
Married or in Civil Partnership	166		
Separated or Divorced	31		
Widowed	166		
Number of times fallen		27.37	0.197
1-5	234		
6-10	12		
11-30	3		
Total ADL's find Difficult		26.71	0.009
0-3	228		

4-6	52		
Total Comorbidities		19.58	0.012
0-2	276		
3-4	5		
Limiting long-standing illness	257	14.99	<0.001
Physical Activity		13.59	0.03
Sedentary	46		
Low	45		
Moderate	23		
High	8		
Age		13.33	0.038
60-69	55		
70-79	127		
80-89	156		
90-99	11		
CASP-19 Total Score		13.10	0.001
0-29	32		
30-57	219		
Alcohol Consumption		11.56	0.48
More than twice a day, daily or almost daily	58		
3-6 times/week	14		
1-2 times/week	64		
1-2 times/month	27		
Once every couple months	15		
1-2 times/year	44		
None in the last 12 months	69		
Ethnicity		9.97	0.01
White	330		
Non-White	5		
Sex		8.68	0.01
Male	129		
Female	255		
Had a fall	238	8.14	0.02
Member of an organisation	89	8.14	0.02
Depression/manic depression	7	4.64	0.10
BMI		4.35	0.361
18.5-24.9	25		
25-29.9	25		
30+	16		
Diseases of the MSK System	104	3.89	0.14
Diseases of the circulatory system	17	3.56	0.17
Smoking Status		2.70	0.26
Current smoker	21		
Stopped smoking	73		
Neoplasms	14	2.47	0.29
Diseases of the respiratory	33	1.58	0.45

system			
Mental disorders	3	0.99	0.61
Metabolic diseases	19	0.39	0.82

eTable 14 Kappa Agreements for Sensitivity Analysis for Missing Data in Trajectory Models

		Kappa*	Agreement (%)	p-value
Overall Population	1st 5 years only	0.8	88.14	<0.001
	Complete cases only	0.68	80.78	<0.001
Hip Fracture Population	1st 5 years only	0.84	90.89	<0.001
	Complete cases only	0.35	60.66	<0.001

* Kappa agreement for group classification
